# Feline umbilical cord-derived mesenchymal stem cells: isolation, identification, and antioxidative stress role through NF-κB signaling pathway

**DOI:** 10.3389/fvets.2023.1203012

**Published:** 2023-05-25

**Authors:** Zhu-Hui Zhai, Jun Li, Zhao You, Yang Cai, Jie Yang, Jie An, Di-Peng Zhao, He-Jie Wang, Min-Min Dou, Rong Du, Jian Qin

**Affiliations:** ^1^College of Veterinary Medicine, Shanxi Agricultural University, Taigu, Shanxi, China; ^2^College of Life Science, Shanxi Agricultural University, Taigu, Shanxi, China; ^3^Center of Experiment Teaching, Shanxi Agricultural University, Taigu, Shanxi, China

**Keywords:** feline, umbilical cord-derived mesenchymal stem cells, fibroblasts, oxidative stress, NF-κB signaling pathway

## Abstract

At present, the differentiation potential and antioxidant activity of feline umbilical cord-derived mesenchymal stem cells (UC-MSCs) have not been clearly studied. In this study, feline UC-MSCs were isolated by tissue adhesion method, identified by flow cytometry detection of cell surface markers (CD44, CD90, CD34, and CD45), and induced differentiation toward osteogenesis and adipogenesis *in vitro*. Furthermore, the oxidative stress model was established with hydrogen peroxide (H_2_O_2_) (100 μM, 300 μM, 500 μM, 700 μM, and 900 μM). The antioxidant properties of feline UC-MSCs and feline fibroblasts were compared by morphological observation, ROS detection, cell viability via CCK-8 assay, as well as oxidative and antioxidative parameters via ELISA. The mRNA expression of genes related to NF-κB pathway was detected via quantitative real-time polymerase chain reaction, while the levels of NF-κB signaling cascade-related proteins were determined via Western Blot. The results showed that feline UC-MSCs highly expressed CD44 and CD90, while negative for CD34 and CD45 expression. Feline UC-MSCs cultured under osteogenic and adipogenic conditions showed good differentiation capacity. After being exposed to different concentrations of H_2_O_2_ for eight hours, feline UC-MSCs exhibited the significantly higher survival rate than feline fibroblasts. A certain concentration of H_2_O_2_ could up-regulate the activities of SOD2 and GSH-Px in feline UC-MSCs. The expression levels of *p50*, *MnSOD*, and *FHC* mRNA in feline UC-MSCs stimulated by 300 μM and 500 μM H_2_O_2_ significantly increased compared with the control group. Furthermore, it was observed that 500 μM H_2_O_2_ significantly enhanced the protein levels of p-IκB, IκB, p-p50, p50, MnSOD, and FHC, which could be reversed by BAY 11-7,082, a NF-κB signaling pathway inhibitor. In conclusion, it was confirmed that feline UC-MSCs, with good osteogenesis and adipogenesis abilities, had better antioxidant property which might be related to NF-κB signaling pathway. This study lays a foundation for the further application of feline UC-MSCs in treating the various inflammatory and oxidative injury diseases of pets.

## 1. Introduction

Mesenchymal stem cells (MSCs) are a kind of adult stem cells with the potential of self-renewal and multi-directional differentiation, which exist in bone marrow, fat, amniotic membranes, placenta, umbilical cord, and other tissues, and can differentiate into osteoblasts, chondrocytes, adipocytes, and nerve cells under appropriate conditions (1, 2). In addition, MSCs were testified to exert antioxidant properties in animal models of various diseases such as chemotherapy-induced injury to the lungs, brain, and aorta; diabetic injuries to the kidney, neurons, and bone formation; gastrointestinal inflammation and aging (3). In recent years, MSCs have been widely used in tissue repair, immune regulation, antioxidation, and other fields in human (4–6). The cat is a popular pet and an important model animal, so it is important to explore the biological characteristics and function of feline MSCs.

At present, feline MSCs have been reported in clinical trials for the treatment of chronic gingival stomatitis, chronic intestinal disease, asthma, kidney disease and other diseases in cats, but mainly focused on fat and bone marrow sources (7). Some studies have shown that feline adipose-derived mesenchymal stem cells (AD-MSCs) face problems such as cell proliferation stagnation during passage, thus hindering the large-scale expansion of autologous MSCs in feline patients (8). Trzil showed that although the injection of feline adipose-derived MSCs was beneficial for feline asthma, the benefits were delayed in time compared to rodent asthma models (9). In acute ischemic kidney injury models, no improvement in renal function was observed by the injection of allogeneic feline bone marrow-derived mesenchymal stem cells (BM-MSCs) (10). Therefore, more studies are needed for further improvement and optimization. Umbilical cord-derived mesenchymal stem cells (UC-MSCs) have the advantages of convenient source, low acquisition cost, without ethical violations, and no damage to animals (11). However, only two reports on the isolation, identification, and characteristic of feline UC-MSCs were found (12, 13). Many aspects such as the antioxidant capacity and mechanism are not clear for feline UC-MSCs, so further research is necessary for expanding the theoretical knowledge and application potential of it.

The antioxidative mechanism of MSCs involves in inhibiting the production and release of intracellular reactive oxygen species (ROS) through upregulating the endogenous antioxidant enzymes, such as superoxide dismutase (SOD), catalase (CAT), and glutathione peroxidase (GSH-Px) or activating the related signaling pathway under oxidative stress (3). Nuclear factor-κB (NF-κB) pathway plays an important role in oxidative stress, immune function, inflammatory response, apoptosis, and cell survival (14–16). Some studies have shown that the addition of H_2_O_2_ to cell culture medium can activate NF-κB activity in several cell lines (17). Under normal circumstances, IκB retains NF-κB in the cytoplasm in an inhibitory state, while when external stimuli such as ultraviolet radiation, lipopolysaccharide or H_2_O_2_ trigger IκB phosphorylation and proteasomal degradation, the free NF-κB dimers were released and translocated to the nucleus, where they bind to the specific DNA sequences and promote the transcription of multiple downstream target genes (18). Among them, manganese superoxide dismutase (MnSOD) and ferritin heavy chain (FHC) are main genes in antioxidant defense (19). Currently, the antioxidant mechanism of NF-κB signaling pathway in H_2_O_2_-treated MSCs is not very clear, especially no relevant reports have been found in UC-MSCs.

Therefore, the purpose of this study was to isolate and identify feline UC-MSCs, and explore the role of NF-κB pathway in their antioxidant stress, which will lay a foundation for revealing the antioxidant mechanism of UC-MSCs, and provide a theoretical basis for the clinical application of feline UC-MSCs.

## 2. Materials and methods

### 2.1. Animals and cell lines

The feline umbilical cord tissues were obtained from female cats after parturition at the small animal clinic in Taigu. Owners agreed to the collection of tissues and all experimental protocols were approved by the Committee for the Care and Use of Experimental Animals, Shanxi Agricultural University, Shanxi, China.

C3H10T1/2 (JCRB Cat# IFO50415, RRID:CVCL_0190) and feline fibroblasts were cultured in Dulbecco’s Modified Eagle Medium (DMEM, Viva Cell, China) containing 20% fetal bovine serum (FBS, Viva Cell, China), 1% penicillin–streptomycin (P/S, 100×, Solarbio, China), 0.1 μM dexamethasone (Solarbio, China), and 55 μM β-mercaptoethanol (Sigma, United States).

### 2.2. Isolation and culture of feline UC-MSCs

The umbilical cord tissue of approximately 2–3 cm length was immersed in sterile Hanks’ balanced salt solution at 4°C, supplemented with P/S, and then immediately transferred to the laboratory. The surface of umbilical cord was rinsed with Dulbecco’s phosphate-buffered saline (DPBS, Sigma, United States) to remove as much blood as possible. The umbilical arteries and veins were removed from each umbilical cord and the Wharton’s jelly (WJ) was carefully separated. The WJ was cut with a scalpel into 0.1–0.5 mm^3^ pieces and transferred to 10 cm tissue culture dishes (Corning, United States) at 0.5–1 cm intervals. The pellet of tissue was then incubated at 37°C in α-modified minimum essential medium (α-MEM, Viva Cell, China) with 20% FBS, 1% P/S, 55 μM β-mercaptoethanol, and 0.1 μM dexamethasone for 2 weeks. During this period, half of the culture medium was refreshed every 7 d until the cells migrated from the fragments borders and reached approximately 80% confluency. Then the cells were harvested with 0.25% Trypsin-EDTA solution (Boster, China) and further passaged. All experiments were performed in cells of passage 3.

### 2.3. Flow cytometry analysis

For fluorescence-activated cell sorting (FACS) analysis, 1 × 10^6^ cells from feline UC-MSCs were used to detect the expression of cell surface markers CD44, CD90, CD34, and CD45. The cells were trypsinized, counted and centrifuged (1,000 rpm/min for 5 min), and washed twice with DPBS. Information of antibodies used for staining is shown in Table 1. 5 μL of each antibody solution was added to 100 μL of cell suspension. Cells were incubated at room temperature in the dark for 20–30 min, then washed twice with DPBS, vortexed, and centrifuged again (1,000 rpm/min for 5 min). Finally, cells were resuspended in 1 mL of DPBS for FACS analysis. At least 10,000 events were recorded for each sample and analyzed with a FC 500 flow cytometer (Beckman, United States). Kaluza Analysis software (Beckman, United States) was used for FACS data analysis.

**Table 1:** Data on antibodies used for FACS analysis.

Cell surface marker	Conjugation	Antibody clone	Isotype	Catalog number	Source	RRID
CD44	PE	IM7	Rat IgG2b	103,023	Biolegend USA	*AB_493686*
CD90	PE	5E10	Mouse IgG1	328,109	Biolegend USA	*AB_893442*
CD34	FITC	581	Mouse IgG1	343,503	Biolegend USA	*AB_1731923*
CD45	FITC	HI30	Mouse IgG1	304,005	Biolegend USA	*AB_314393*

### 2.4. *In vitro* mesodermal differentiation

#### 2.4.1. Osteogenesis

Three types of cells (C3H10T1/2, feline UC-MSCs, and feline fibroblasts) were seeded into 6-well plates (Corning, United States) at a density of 5 × 10^5^ cells/well. The original culture medium was discarded the second day, and the osteogenic medium consisting of DMEM supplemented with 20% FBS, 1% P/S, 55 μM β-mercaptoethanol, 0.1 μM dexamethasone, 2 mM β-glycerophosphate (Wako, 048-34,332, JPN), and 0.1 mM L-ascorbic acid phosphate (Wako, 013-12,061, JPN) was added for induction. As a negative control, cells were cultured in a normal medium deprived of the differentiation factors. Cells were cultured for 14–21 days and differentiation medium was changed every 2 days. At the end of the differentiation period cells were stained with Alizarin Red (1%, pH 4.2, Solarbio, China) for 30 min and imaged in an inverted fluorescence microscope (Leica, Germany) and the differences among the three types of cells were assessed visually.

#### 2.4.2. Adipogenesis

Three types of cells (C3H10T1/2, feline UC-MSCs, and feline fibroblasts) were seeded into 6-well plates at a density of 5 × 10^5^ cells/well. The original medium was discarded when the cells reached 90% of confluence. The adipogenic medium consisting of DMEM supplemented with 20% FBS, 1% P/S, 55 μM β-mercaptoethanol, 0.1 μM dexamethasone, 0.1 mM L-ascorbic acid phosphate, 0.5 mM 3-Isobutyl-1-methylxanthine (Sigma, I5879, United States), 60 μM indomethacin (Sigma, I7378, United States) and 10 μg/mL insulin (Solarbio, I8830, China) was added into the cells. As a negative control, cells were cultured in a normal medium deprived of the differentiation factors. Cells were cultured for 7–21 days and differentiation medium was changed every 2–3 days. When the lipid droplets in the cytoplasm were observed, the medium was carefully discarded. The cells were washed with DPBS twice, fixed in 4% paraformaldehyde (PFA, Solarbio, China) for 20 min, stained with Oil red O (Solarbio, China) for 30 min, and photographed under an inverted fluorescence microscope. The differences among the three types of cells were assessed visually.

#### 2.4.3. Optimization for osteogenic and adipogenic differentiation of feline UC-MSCs

Feline UC-MSCs were seeded into 6-well plates at a density of 5 × 10^5^ cells/well. When the cells achieved appropriate confluency, osteogenic, and adipogenic differentiation were induced according to the optimized protocols: (1) The optimal osteogenic medium consisting of α-MEM supplemented with 20% FBS, 1% P/S, 55 μM β-mercaptoethanol, 0.1 μM dexamethasone, 10 mM β-glycerophosphate, and 0.2 mM L-ascorbic acid phosphate. (2) The optimal adipogenic medium consisting of α-MEM supplemented with 20% FBS, 1% P/S, 55 μM β-mercaptoethanol, 10 μM dexamethasone, 0.1 mM L-ascorbic acid phosphate, 0.5 mM 3-Isobutyl-1-methylxanthine, 60 μM indomethacin, and 10 μg/mL insulin. After 2 weeks, osteogenic differentiation was detected by Alizarin Red staining while adipogenic differentiation was detected by Oil Red O staining. Differentiated cells were then photographed under an inverted fluorescence microscope.

### 2.5. Establishment of the oxidative stress model for feline UC-MSCs and feline fibroblasts

#### 2.5.1. *In vitro* model of H_2_O_2_-induced oxidative stress

Feline UC-MSCs and feline fibroblasts were seeded into 6-well plates at a density of 5 × 10^5^ cells/well. When the cells grew to the appropriate confluence, the original medium was replaced and H_2_O_2_ (3%, Sigma, United States) was added at the concentration of 100 μM, 300 μM, 500 μM, 700 μM, and 900 μM to the culture, with a control group being set up at the same time. All groups were cultured for an additional 8 h under constant experimental conditions before analyses.

#### 2.5.2. Cell viability assay

The cell viability of feline UC-MSCs and feline fibroblasts after H_2_O_2_ treatment was evaluated using Cell Counting Kit-8 (CCK-8, Beyotime Biotechnology, China). Cells were seeded into 96-well plates (Corning, United States) at a density of 5 × 10^3^ cells/well. The original medium was replaced and different concentrations of H_2_O_2_ were added for 8 h. Next, 10 μL of CCK-8 solution was added into each well and incubated for 2 h at 37°C, after which the absorbance was measured at 450 nm using a microplate reader (Molecular Devices, United States). The blank control consisted of CCK-8 reagent and complete medium with no cells. Each experiment was repeated four times and the optical density (OD) was calculated as follows:


$$\begin{array}{c} \mathrm{Cell Viability}\;\left(\%\right)=\left(\begin{array}{l} A450\;\mathrm{of the experimental group}\\ –A450\;\mathrm{of the blank group} \end{array}\right)\\ /\left(\begin{array}{l} A450\;\mathrm{of the control group}\\ –A450\;\mathrm{of the blank group} \end{array}\right) \end{array}$$

#### 2.5.3. Detection of intracellular ROS

The levels of intracellular ROS were determined using an ROS assay kit (Beyotime Biotechnology, China) following the manufacturer’s protocol. The peroxide-sensitive fluorescent probe 2,7-dichlorodihydrofluorescein diacetate (DCFH-DA) was used to measure the intracellular levels of ROS in H_2_O_2_-treated feline UC-MSCs and feline fibroblasts. Cells were seeded into 6-well plates at a density of 5 × 10^5^ cells/well and treated with different concentrations of H_2_O_2_. Eight hours later, the cells were harvested and then washed twice with DPBS and incubated with diluted DCFH-DA (10 μmol/L) at 37°C for 30 min in the dark for final analysis by inverted fluorescence microscope. The fluorescence intensity of the cells was quantified via Image-Pro Plus (RRID:SCR_007369) software.

#### 2.5.4. Detection of oxidative and antioxidative parameters

Cells were seeded into 6-well plates at a density of 5 × 10^5^ cells/well. The feline UC-MSCs and feline fibroblasts were treated for 8 h with various concentrations of H_2_O_2_ (100 μM, 300 μM, and 500 μM). Following H_2_O_2_ treatment, cells were collected for the determination of four oxidative/antioxidative biomarkers. The MDA level, and activities of CAT, SOD2, and GSH-Px were detected using commercially available assay kits (Shanghai Enzyme Linked Biotechnology, China) following the manufacturer’s instructions.

### 2.6. Quantitative real-time polymerase chain reaction (qRT-PCR)

Feline UC-MSCs were treated with H_2_O_2_ (100 μM, 300 μM, and 500 μM) alone or together with NF-κB inhibitor BAY 11-7,082 (20 μM, Selleck, United States) for 8 h. Cells were lysed with 1 mL of TRIzol reagent (TaKaRa, JPN), and cDNA was synthesized with the PrimeScript^™^RT reagent Kit (TaKaRa, JPN). The reaction conditions were as follows: 95°C for 30 s, followed by 42 cycles at 95°C for 5 s and at 60°C for 30 s. The gene specific primers were designed with the Primer 5.0 program and NCBI Primer-BLAST (Table 2). The mRNA level of *GAPDH* was determined for the normalization of the *IκB*, *p50*, *MnSOD*, and *FHC* mRNA expression values. Data were quantified using 2^−ΔΔCt^ method.

**Table 2:** Primer sequence information.

Gene name	Primer sequences (5′-3′)	Accession no.	Product (bp)
*GAPDH*	F: TCATCCATGACCACTTCGGC R: AGATCCACGACGGACACATTG	NM_001009307.1	247
*IκB*	F: CCTCGTGTCGCTTTTGTTGA R: CGTCCTCTGTGAACTCTGACTC	XM_003987542.6	203
*P50*	F: TGGCACTGGAGAAGATGAAGTT R: CACTGCGTAGTCAAAAAGGGC	XM_023252904.2	142
*MnSOD*	F: TCACATCAACGCCCAGATCAT R: CCAGCGCCTCTCGATACC	XM_023254547.2	101
*FHC*	F: AACGACCCCCATTTGTGTGA R: GCCATGCCAGATTCGGGAGT	NM_001048151.1	125

### 2.7. Western blot

Cells were collected and lysed using radio immunoprecipitation assay (RIPA) buffer (Beyotime Biotechnology, China). The protein samples were separated with the 10% sodium dodecyl sulfate-polyacrylamide gel and then transferred onto nitrocellulose (NC) membranes (Boster, China). Afterwards, the blocking of membranes was done with 5% skimmed milk for 2 hours and the membranes were inoculated overnight with primary antibodies against Rabbit anti-IκB Alpha (1:2000, Proteintech, United States, Cat#10268-1-AP, RRID:AB_2151423), Rabbit anti-Phospho-IκB Alpha-S32 (1:2000, ABclonal, China), Rabbit anti-NF-κB p50 (1:1000, Proteintech, United States, Cat# 14220-1-AP, RRID:AB_2153393), Rabbit anti-Phospho-NF-κB p50-S337 (1:2000, ABclonal, China), Rabbit anti-MnSOD (1:20000, Proteintech, United States, Cat# 24127-1-AP, RRID:AB_2879437), Rabbit-anti-FHC (1:500, Boster, China), and Rabbit anti-GAPDH (1:20000, Proteintech, United States, Cat# 10494-1-AP, RRID:AB_2263076) in TBST at 4°C. The membranes were then washed and incubated with goat anti-rabbit HRP-conjugated secondary antibody (1:1000, Solarbio, China, Cat# SE134, RRID:AB_2797593) at room temperature for 1 h. The protein bands were visualized with an ECL reagent kit (Boster, China). The densities of protein blots were quantified by using Image J software (NIH, Bethesda, United States, RRID:SCR_003070) and normalized to the level of GAPDH.

### 2.8. Statistical analysis

Statistical differences were analyzed using the SPSS software version 21 (IBM Corp., Armonk, United States, RRID:SCR_019096), and GraphPad Prism 9 (San Diego, United States, RRID:SCR_002798). The comparison between two or more groups was conducted by *t*-test or one-way analysis of variance (ANOVA). Data were expressed as mean ± standard deviation (SD) and *p* < 0.05 was considered as statistically significant.

## 3. Results

### 3.1. Isolation and proliferation of feline UC-MSCs

**Figure 1:**
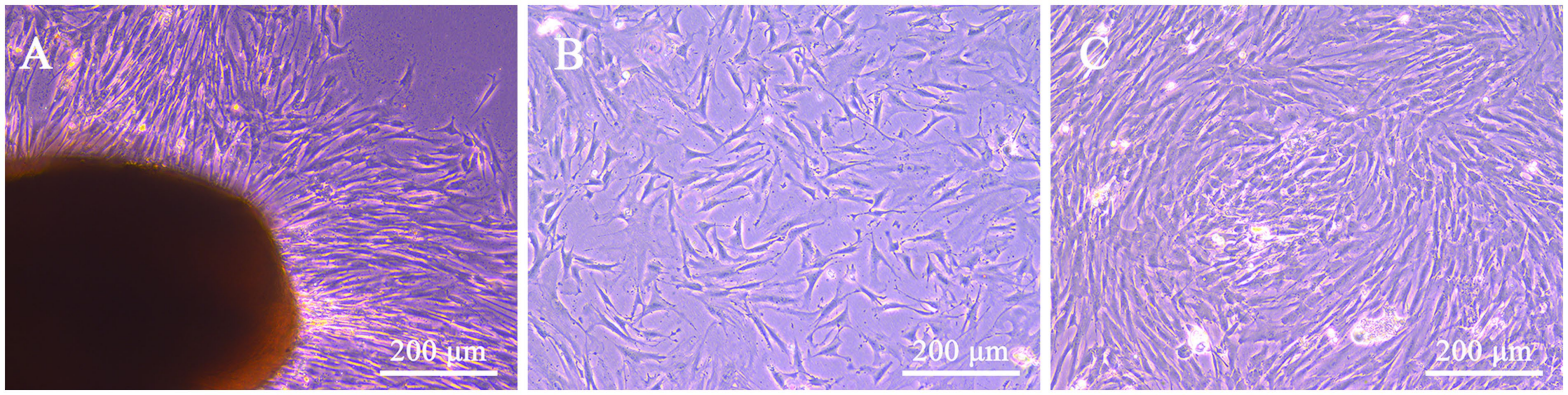
Morphological characteristics of feline UC-MSCs isolated and cultured with the tissue adhesion method. Feline UC-MSCs migrated from adherent tissue on the tenth day **(A)**. Morphology of feline UC-MSCs after passaging for 48 h **(B)**. Morphological characteristics of the third-generation feline UC-MSCs **(C)**. Scale bar: 200 μm.

At day 10 of primary culture, a small number of adhered cells could be observed migrating from the boundary of WJ tissue fragments from feline umbilical cord, and exhibited a long spindle shape similar to fibroblasts (Figure 1A). After passaging, the proliferation rate increased exponentially, and the cells reached 80% confluence on the second day (Figure 1B). With the passaging of the cells, the cell morphologies were gradually uniform, which were consistent with the characteristics of MSCs (Figure 1C).

### 3.2. Expression of feline UC-MSCs phenotypical markers

**Figure 2:**
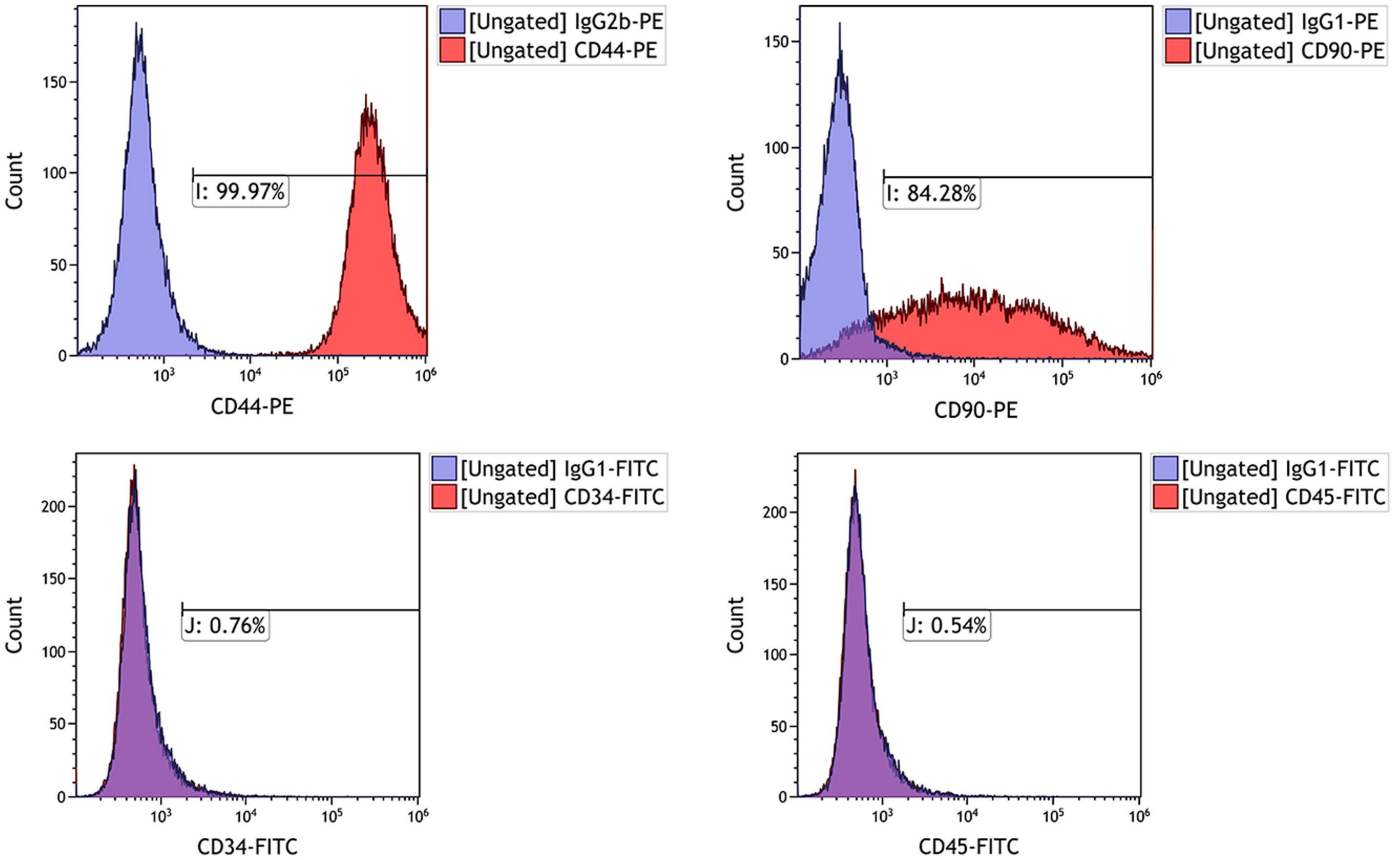
Analysis by flow cytometry of the expression levels of cell surface markers CD44, CD90, CD34, and CD45 in feline UC-MSCs. The purple histograms represent isotype controls and red histograms represent cell surface marker staining.

To confirm that the isolated and cultured cells from feline umbilical cord tissue corresponded to MSCs, we tested the expression of several MSCs phenotypical markers including CD44, CD90, CD34, and CD45. FACS analysis revealed that feline UC-MSCs were prominently positive for CD44 and CD90, and negative for hematopoietic lineage markers including CD34 and CD45 (Figure 2).

### 3.3. Mesodermal differentiation potential of feline UC-MSCs

**Figure 3:**
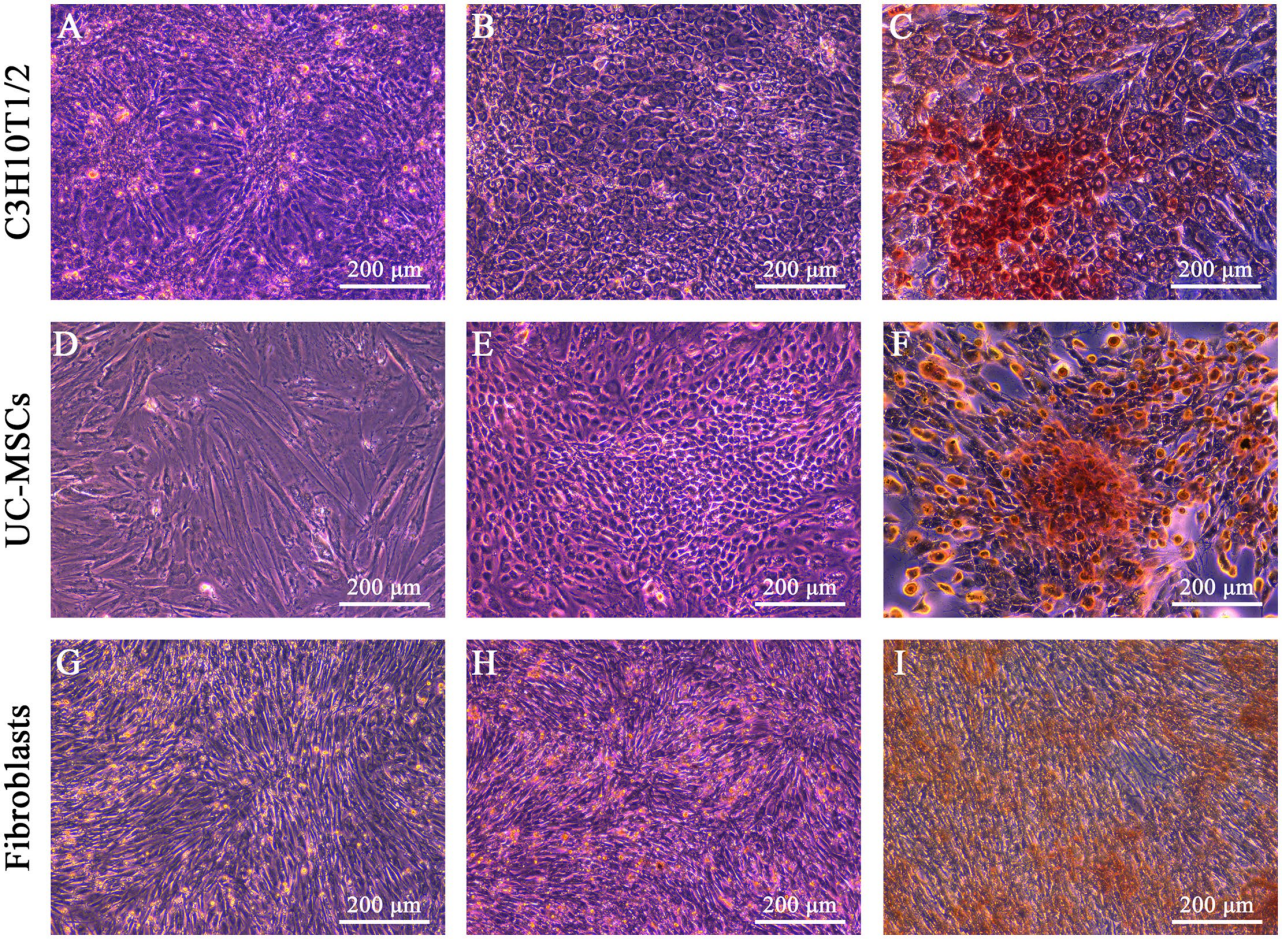
Osteogenic differentiation of three types of cells. No osteogenic cells were detected in C3H10T1/2 **(A)**, feline UC-MSCs **(D)**, and feline fibroblasts **(G)** under the uninduced control treatment. Morphological changes were detected in C3H10T1/2 **(B)**, feline UC-MSCs **(E)**, and feline fibroblasts **(H)** during osteogenesis. Images of Alizarin red staining in C3H10T1/2 **(C)**, feline UC-MSCs **(F)**, and feline fibroblasts **(I)**. Scale bar: 200 μm.

We evaluated the morphological characters of three types of cells after osteogenic and adipogenic induction, here C3H10T1/2 being the positive control while feline fibroblasts being the negative control. After osteogenic induction, the shapes of C3H10T1/2 and feline UC-MSCs changed from fibrous shapes to polygons and scales (Figures 3B,E). In the control wells without osteogenic induction, C3H10T1/2, feline UC-MSCs, and feline fibroblasts only showed an increase in density (Figures 3A,D,G). All three types of cells were stained with Alizarin Red, and the red calcium nodules appeared in C3H10T1/2 and feline UC-MSCs after 14 and 21 days of induction, respectively (Figures 3C,F). Less accumulation of calcium nodules in feline UC-MSCs was observed compared to C3H10T1/2, which suggested that the osteogenic induction condition for feline UC-MSCs needed to be optimized. However, feline fibroblasts did not change in morphology (Figure 3H), and no calcium nodules were observed after staining procedure (Figure 3I).

**Figure 4:**
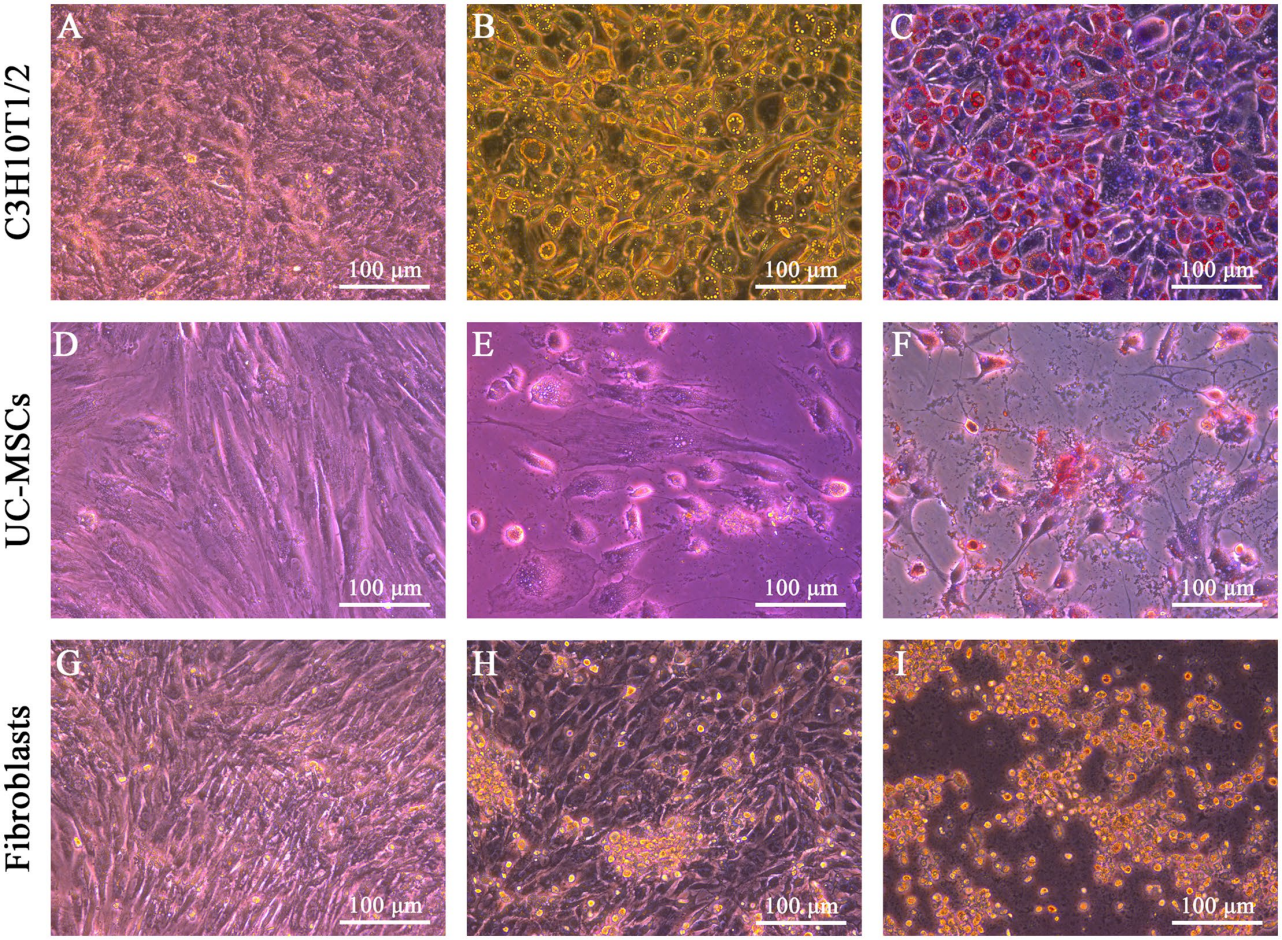
Adipogenic differentiation of three types of cells. No adipogenic cells were detected in C3H10T1/2 **(A)**, feline UC-MSCs **(D)**, and feline fibroblasts **(G)** under the uninduced control treatment. Morphological changes were detected in C3H10T1/2 **(B)**, feline UC-MSCs **(E)**, and feline fibroblasts **(H)** during adipogenesis. Images of Oil red O staining in C3H10T1/2 **(C)**, and feline UC-MSCs **(F)**, and negative results of adipogenic differentiation in feline fibroblasts **(I)**. Scale bar: 100 μm.

After adipogenic induction, lipid droplets were observed in C3H10T1/2 and feline UC-MSCs at the 6th and 9th day, respectively (Figures 4B,E). In the control wells without adipogenic induction, C3H10T1/2, feline UC-MSCs, and feline fibroblasts only showed an increase in density (Figures 4A,D,G). Oil red O staining results showed that the red lipid droplets were clearly observed in C3H10T1/2 and feline UC-MSCs after 7 and 21 days of induction, respectively (Figures 4C,F). Less accumulation of lipid droplets in feline UC-MSCs appeared compared to C3H10T1/2, which suggested that the adipogenic induction condition for feline UC-MSCs needed to be optimized. In feline fibroblasts, more and more cells died and no lipid droplets appeared during the adipogenic induction (Figures 4H,I).

### 3.4. Osteogenic and adipogenic differentiation of feline UC-MSCs in optimized conditions

**Figure 5:**
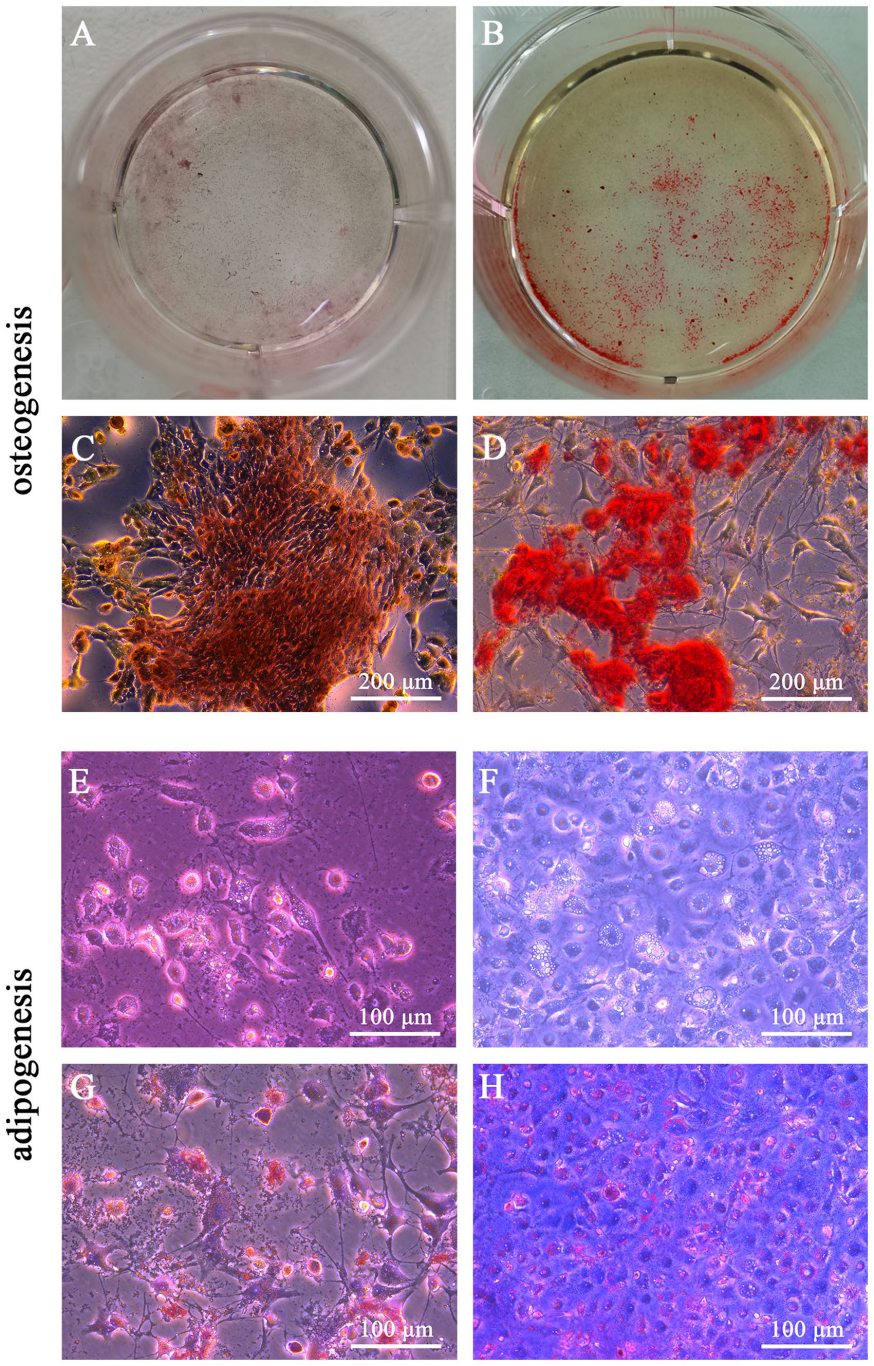
Optimization for osteogenic and adipogenic differentiation of feline UC-MSCs. Comparison of Alizarin red staining areas in feline UC-MSCs under previous conditions **(A)** and optimized conditions **(B)**. Comparison of calcium nodules in feline UC-MSCs under previous conditions **(C)** and optimized conditions **(D)**. Comparison of the accumulation of lipid droplets in feline UC-MSCs under previous conditions **(E)** and optimized conditions **(F)**. Comparison of lipid droplets staining in feline UC-MSCs under previous conditions **(G)** and optimized conditions **(H)**. Scale bar: 100 μm, 200 μm.

When the β-glycerophosphate and L-ascorbic acid phosphate concentration further increased, the results of Alizarin Red staining on day 14 showed that the calcium nodules were more clustered, compact, and numerous (Figures 5B,D) than before optimization (Figures 5A,C), which suggested the optimized osteogenic medium significantly enhanced the osteogenesis of feline UC-MSCs and reduced the induction time from 21 days to 14 days.

We optimized the adipogenic medium based on the increase of dexamethasone concentration and compared the lipid accumulation of feline UC-MSCs after adipogenic induction. As shown in Figures 5F,H, the lipid droplets were more numerous and clearer than before optimization (Figures 5E,G). Moreover, the optimized adipogenic medium reduced the induction time from 21 days to 14 days.

### 3.5. H_2_O_2_-induced morphological changes of feline UC-MSCs and feline fibroblasts

**Figure 6:**
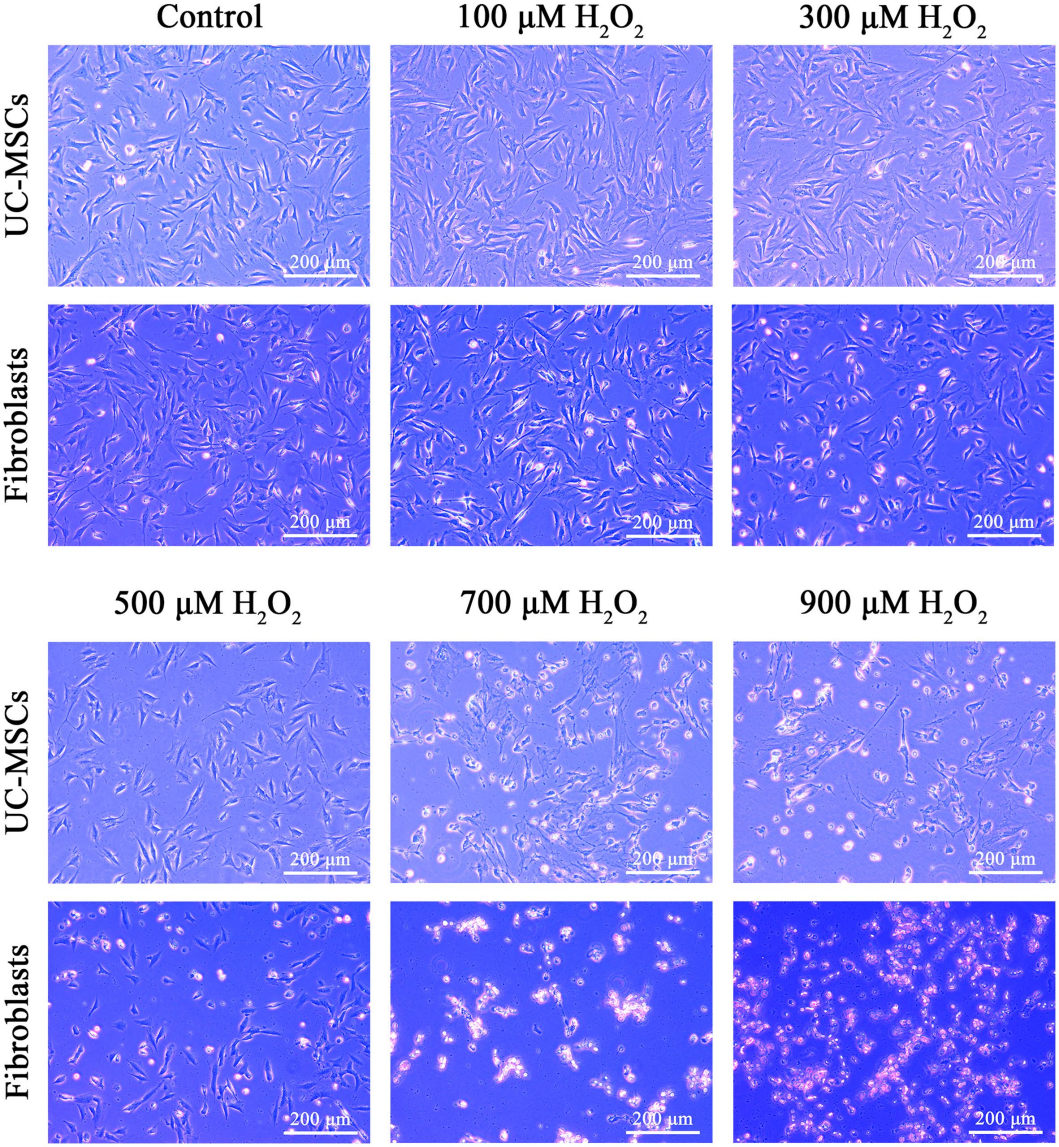
Effects of different concentrations of H_2_O_2_ on cell morphology in feline UC-MSCs and feline fibroblasts. Scale bar: 200 μm.

As shown in Figure 6, the morphology of feline fibroblasts and feline UC-MSCs did not show obvious change in 100 μM H_2_O_2_-treated group. When the concentration of H_2_O_2_ was up to 300 μM, feline fibroblasts were wrinkled, damaged, and loosely arranged, and lacked a fibrous shape. Plenty of feline fibroblasts were detached and floated in culture media and the number of cells was significantly reduced in 500 μM, 700 μM, and 900 μM H_2_O_2_-treated groups. By comparison, feline UC-MSCs treated by 300 μM H_2_O_2_ displayed a spindle shape, which did not show obvious morphological change. Although feline UC-MSCs showed changes such as shrinking, irregular morphology and uneven size when the concentration of H_2_O_2_ was up to 500 μM, 700 μM, and 900 μM, some cells remained alive. The results demonstrated that feline UC-MSCs had higher resistance to H_2_O_2_ compared with feline fibroblasts.

### 3.6. Effects of H_2_O_2_ on cell viability in feline UC-MSCs and feline fibroblasts

**Figure 7:**
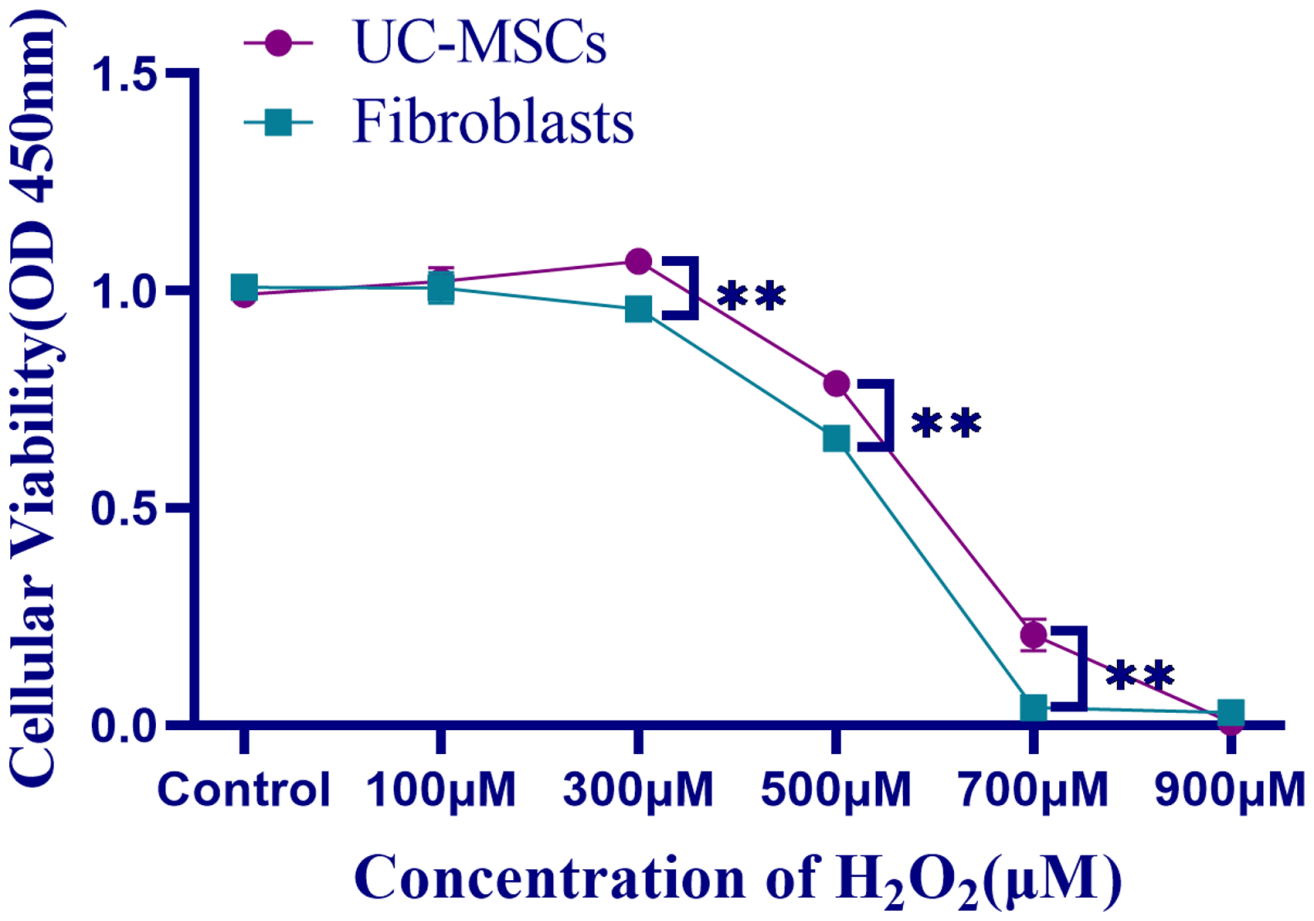
Effects of different concentrations of H_2_O_2_ on cell viability in feline UC-MSCs and feline fibroblasts. All data are presented as mean ± SD and *n* = 4 in each group. **p < 0.01.

The effects of H_2_O_2_ on feline UC-MSCs and feline fibroblasts viability were shown in Figure 7. The cell survival rate of feline UC-MSCs was significantly higher than that of feline fibroblasts in 300 μM, 500 μM, and 700 μM H_2_O_2_-treated group (*p* < 0.01). When the concentration of H_2_O_2_ was up to 700 μM or 900 μM, the survival rate of feline fibroblasts or feline UC-MSCs decreased to nearly zero, respectively. Therefore, the treatment with the concentration of 100 μM, 300 μM, and 500 μM H_2_O_2_ for 8 h was chosen as the model condition of oxidative stress for subsequent studies.

### 3.7. Effects of H_2_O_2_ on ROS level in feline UC-MSCs and feline fibroblasts

**Figure 8:**
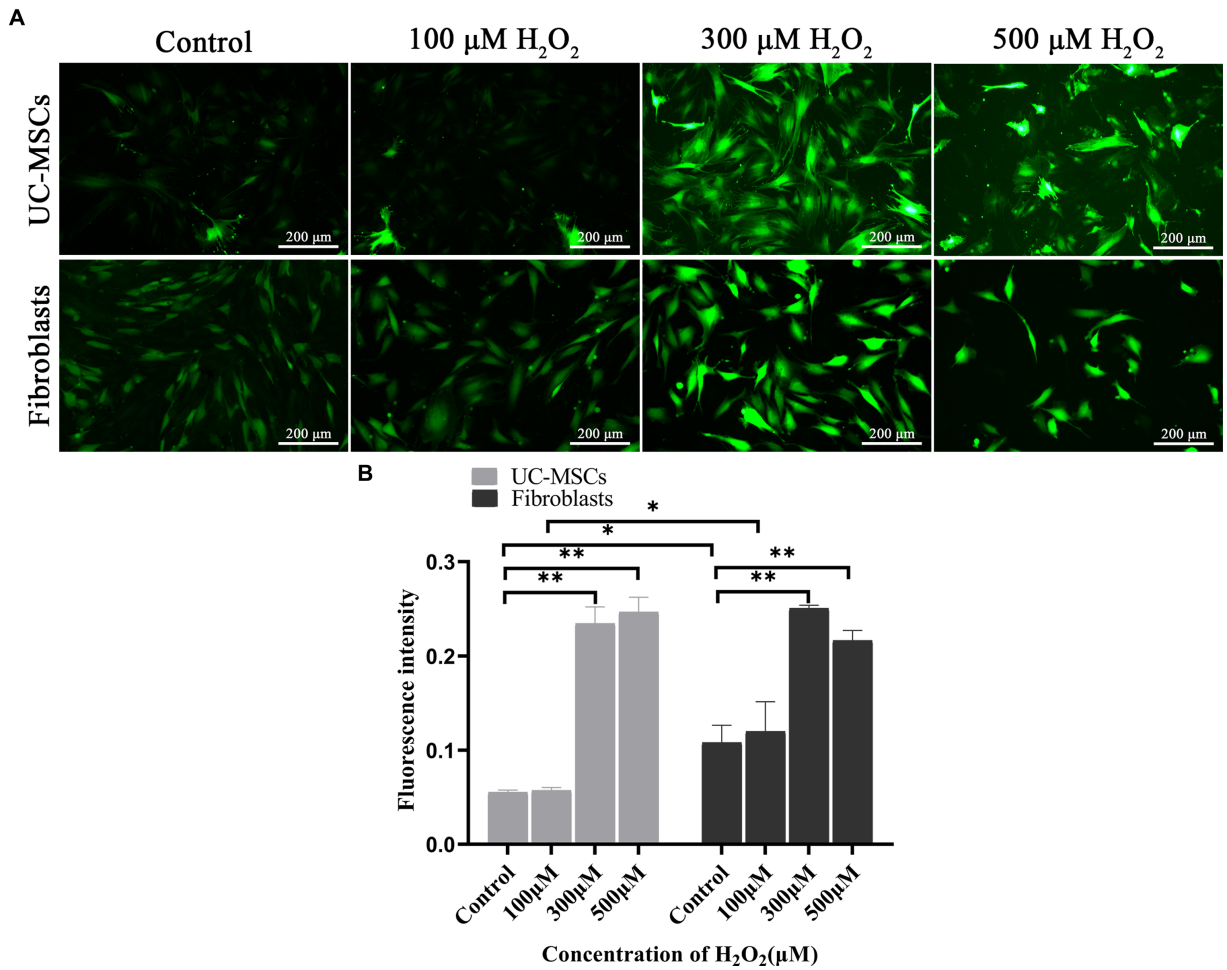
Effects of different concentrations of H_2_O_2_ on ROS production in feline UC-MSCs and feline fibroblasts. **(A)** Observation of ROS fluorescence. Scale bar: 200 μm. **(B)** Quantitative analysis of the mean fluorescence intensity (MFI). All data are presented as mean ± SD and *n* = 3 in each group. *p < 0.05, **p < 0.01.

The intracellular ROS change tendency was similar between feline UC-MSCs and feline fibroblasts, but the ROS level in feline UC-MSCs was lower than that in feline fibroblasts in the control group and 100 μM H_2_O_2_ group (*p* < 0.05) (Figure 8A). Compared with the control group, after treated with 300 μM and 500 μM of H_2_O_2_ for 8 h, the ROS content significantly increased both in feline UC-MSCs and feline fibroblasts (*p* < 0.01) (Figure 8B).

### 3.8. Effects of H_2_O_2_ on the activities of CAT, SOD2, GSH-Px and level of MDA in feline UC-MSCs and feline fibroblasts

**Figure 9:**
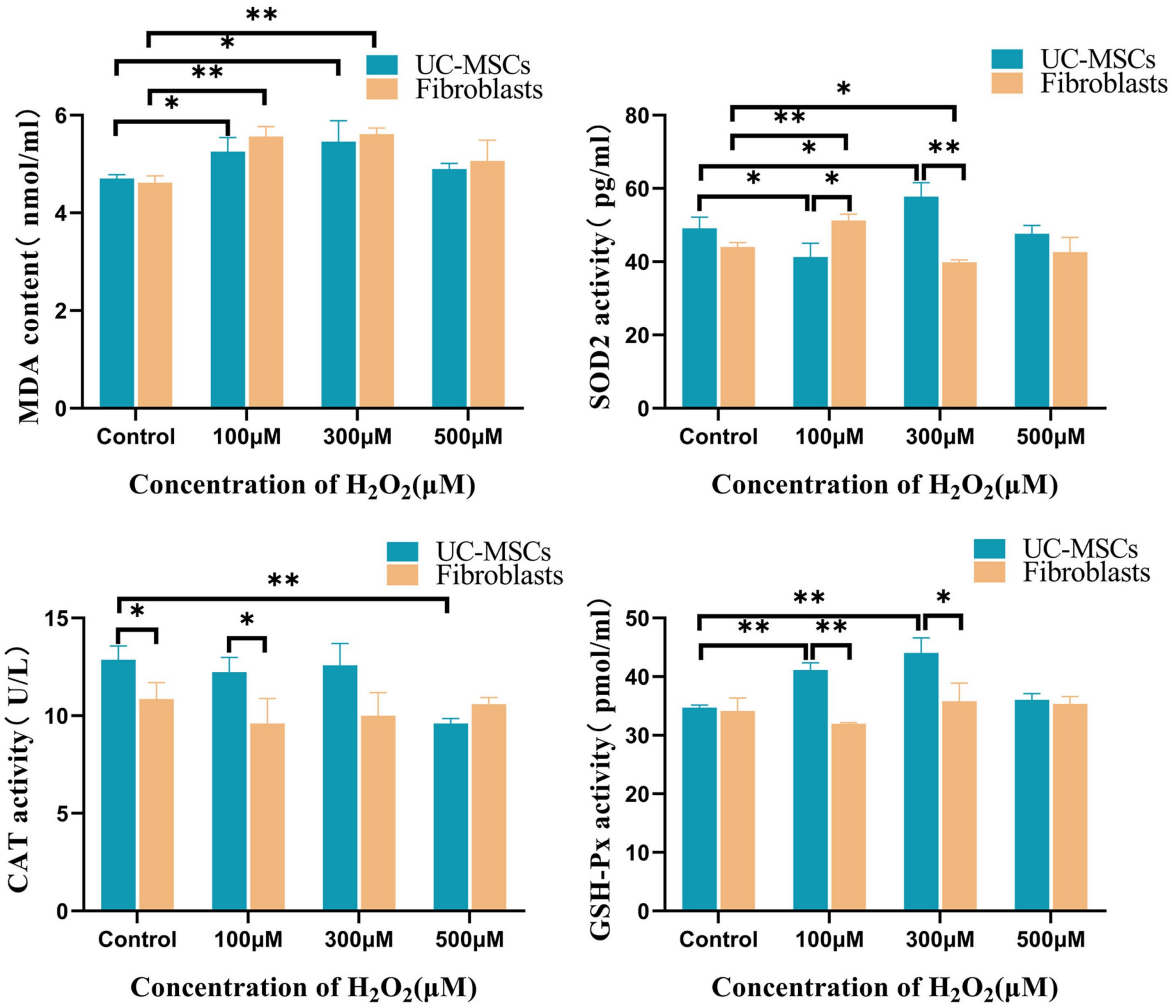
Effects of different concentrations of H_2_O_2_ on intracellular MDA, CAT, SOD2, and GSH-Px levels in feline UC-MSCs and feline fibroblasts. All data are presented as mean ± SD and *n* = 3 in each group. *p < 0.05, **p < 0.01.

As shown in Figure 9, compared to the corresponding control group, after treated with 100 μM and 300 μM of H_2_O_2_ for 8 h, the MDA level significantly increased by 11.71 and 16.06% in feline UC-MSCs (*p* < 0.05), and by 20.38 and 21.47% in feline fibroblasts (*p* < 0.01), respectively. Compared to the control group, after feline UC-MSCs were treated with 500 μM H_2_O_2_, the CAT activity significantly decreased by 25.36% (*p* < 0.01). While in the control group and 100 μM H_2_O_2_-treated group, the CAT activity in feline UC-MSCs was significantly higher than that in feline fibroblasts (*p* < 0.05). Compared to the control group, the SOD2 activity of feline UC-MSCs decreased significantly, and was significantly lower than that of feline fibroblasts in 100 μM H_2_O_2_-treated group (*p* < 0.05). But the SOD2 activity of feline UC-MSCs increased significantly by 17.56% (*p* < 0.05), and was significantly higher than that of feline fibroblasts in 300 μM H_2_O_2_-treated group (*p* < 0.01). Compared to the control group, after feline UC-MSCs were treated with 100 μM and 300 μM of H_2_O_2_, the GSH-Px activity significantly increased by 18.47 and 26.80% (*p* < 0.01), and significantly higher than that in feline fibroblasts (*p* < 0.01 or *p* < 0.05). The activities of CAT and GSH-Px did not change in feline fibroblasts after being treated with 100 μM, 300 μM, and 500 μM H_2_O_2_.

### 3.9. Effects of NF-κB inhibitor on the H_2_O_2_-induced morphological changes of feline UC-MSCs

**Figure 10:**
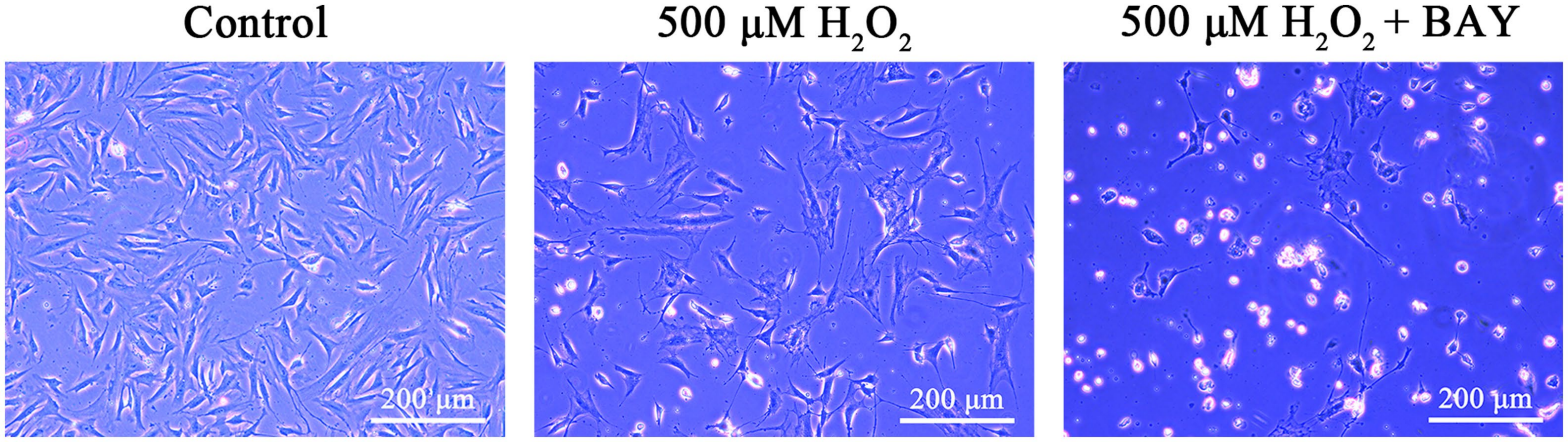
Morphological changes of feline UC-MSCs treated by H_2_O_2_ alone or together with BAY 11-7,082. Scale bar: 200 μm.

We observed the morphological changes of feline UC-MSCs after adding 500 μM H_2_O_2_ alone or together with NF-κB inhibitor BAY 11-7,082. As shown in Figure 10, there were more abnormal even dead cells, appearing cell fragmentation, cell dissolution, cell shrinkage, cell floating, and so on, in 500 μM H_2_O_2_ + BAY 11-7,082 group compared with 500 μM H_2_O_2_-treated group. It suggested that NF-κB signaling pathway was involved in the antioxidative process of feline UC-MSCs responding to H_2_O_2_ stimulus.

### 3.10. Effects of H_2_O_2_ on the mRNA expression levels of NF-κB pathway related genes in feline UC-MSCs

**Figure 11:**
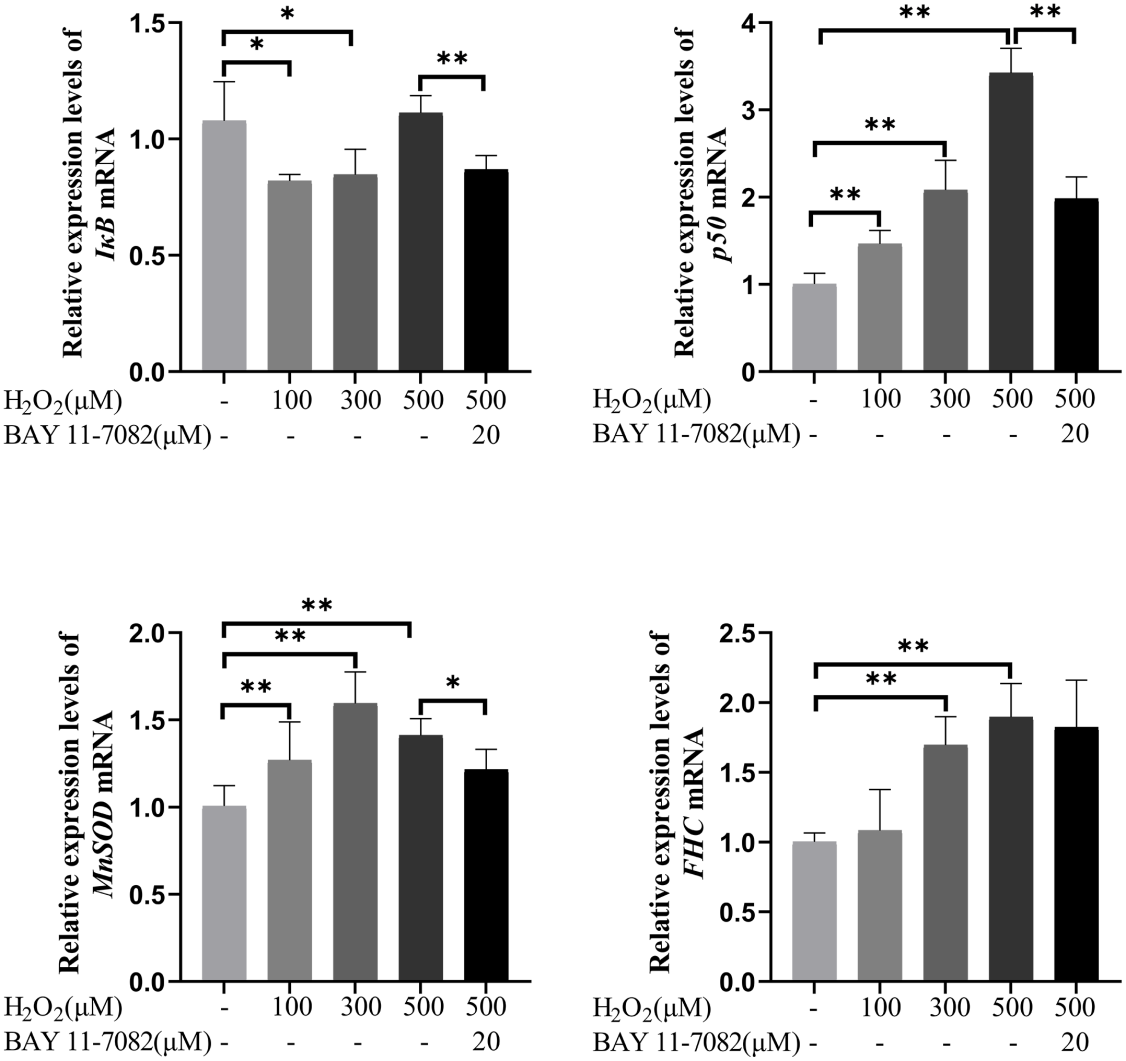
Effects of H_2_O_2_ alone or together with BAY 11-7,082 on mRNA expression of NF-κB pathway-related genes including *IκB*, *p50*, *MnSOD*, and *FHC* in feline UC-MSCs. All data are presented as mean ± SD and *n* = 6 in each group. *p < 0.05, **p < 0.01.

As shown in Figure 11, compared with the control group, the expression of *IκB* mRNA significantly decreased by 23.95 and 21.44% in 100 μM and 300 μM H_2_O_2_-treated group, respectively (*p* < 0.05). The expression of *p*50 mRNA significantly increased by 45.94, 107.40, and 240.98% in 100 μM, 300 μM, and 500 μM H_2_O_2_-treated group, respectively (*p* < 0.01). The expression of *MnSOD* mRNA significantly increased by 26.23, 58.52, and 40.24% in 100 μM, 300 μM, and 500 μM H_2_O_2_-treated group, respectively (*p* < 0.01). The expression of *FHC* mRNA significantly increased by 69.30 and 89.11% in 300 μM and 500 μM H_2_O_2_-treated group, respectively (*p* < 0.01). BAY 11-7,082, an inhibitor of IκB-α phosphorylation, had strong inhibitory effects on NF-κB regulated genes like *IκB*, *p50* and *MnSOD* (*p* < 0.05 or *p* < 0.01).

### 3.11. Effects of H_2_O_2_ on the expression levels of NF-κB pathway related proteins in feline UC-MSCs

**Figure 12:**
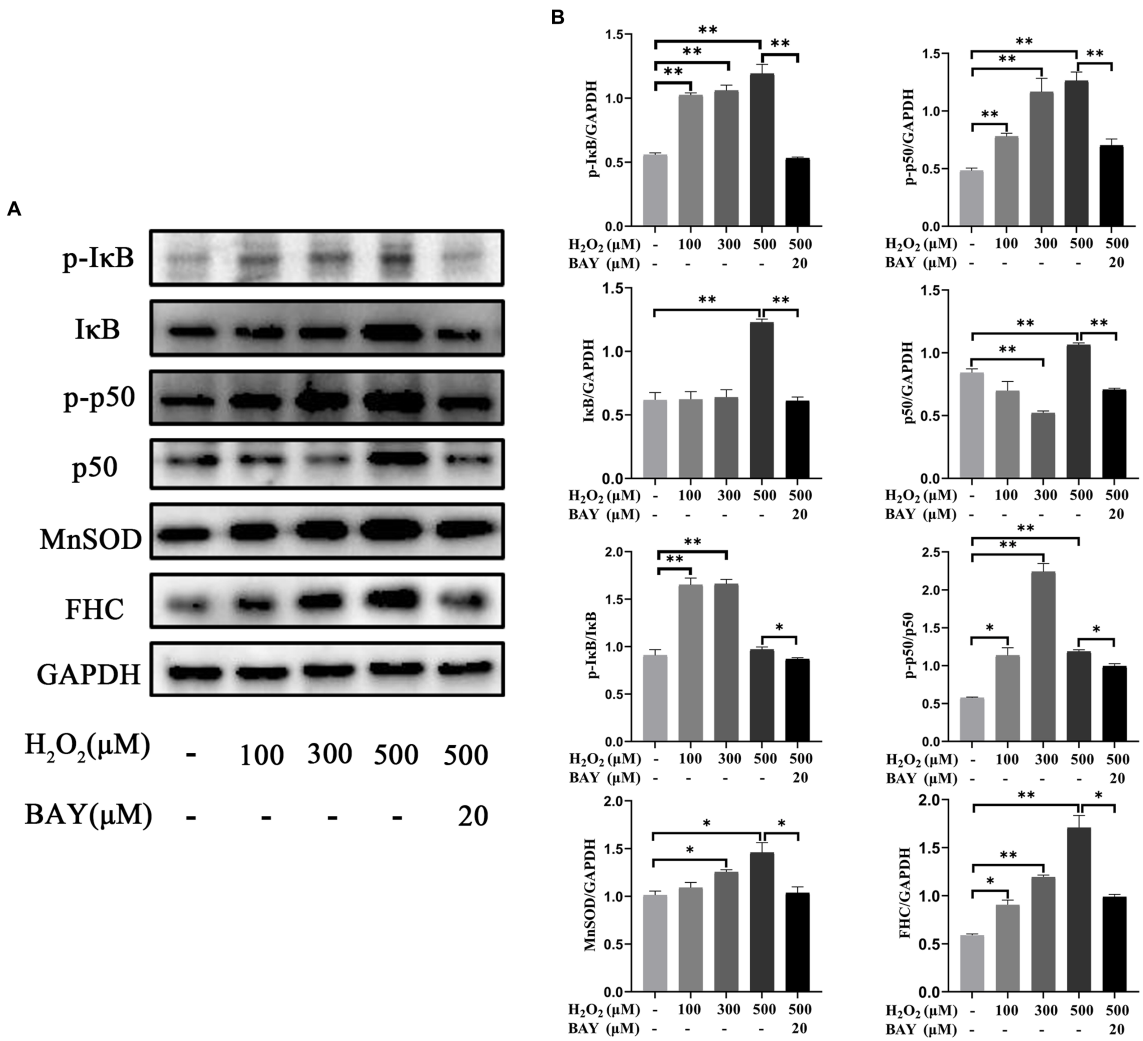
Effects of H_2_O_2_ alone or together with BAY 11-7,082 on NF-κB pathway in feline UC-MSCs. **(A)** Western blot analysis of p-IκB, IκB, p-p50, p50, MnSOD, FHC, and GAPDH. **(B)** Quantitative analysis of p-IκB, IκB, p-IκB/IκB, p-p50, p50, p-p50/p50, MnSOD, and FHC relative levels. All data are presented as mean ± SD and *n* = 3 in each group. *p < 0.05, **p < 0.01.

The expression changes of proteins related to NF-κB pathway were shown in Figure 12. Compared with the control group, the protein levels of p-IκB significantly increased by 83.90, 90.23, and 113.81% in 100 μM, 300 μM, and 500 μM H_2_O_2_-treated group, respectively (*p* < 0.01). The protein levels of IκB significantly increased by 99.07% in 500 μM H_2_O_2_-treated group (*p* < 0.01). The ratio of p-IκB to total IκB significantly increased by 81.70 and 82.82% in 100 μM and 300 μM H_2_O_2_-treated group, respectively (*p* < 0.01). The protein levels of p-p50 significantly increased by 61.19, 141.28, and 161.39% in 100 μM, 300 μM, and 500 μM H_2_O_2_-treated group, respectively (*p* < 0.01). The protein levels of p50 significantly decreased by 38.16% in 300 μM H_2_O_2_-treated group (*p* < 0.01) but increased by 26.44% in 500 μM H_2_O_2_-treated group (*p* < 0.01). The ratio of p-p50 to total p50 increased by 97.67, 289.80, and 106.42% in 100 μM, 300 μM, and 500 μM H_2_O_2_-treated group, respectively (*p* < 0.05 or *p* < 0.01). The protein levels of MnSOD significantly increased by 23.89 and 44.07% in 300 μM, and 500 μM H_2_O_2_-treated group (*p* < 0.05). The protein levels of FHC significantly increased by 53.68, 102.69, and 190.22% in 100 μM, 300 μM, and 500 μM H_2_O_2_-treated group (*p* < 0.05 or *p* < 0.01). The upregulations of these NF-κB signaling pathway-related proteins induced by 500 μM H_2_O_2_ were significantly reversed by the addition of BAY 11-7,082 (*p* < 0.05 or *p* < 0.01).

## 4. Discussion

### 4.1. Surface markers and differentiation potential of feline UC-MSCs

In this study, feline UC-MSCs were isolated successfully and showed characteristics of typical MSCs including fibroblast-like morphology, surface markers and differentiation potential. Although it seemed there were a small number of heterogeneous cells in the isolated feline UC-MSCs from Wharton’s jelly of feline umbilical cord at P0, which because the original feline umbilical cord tissue contains round epithelial cells, they were replaced by long spindle UC-MSCs after passage as previously reported (20). All the feline UC-MSCs could adhere to the bottom of the culture bottle and show an extremely rapid proliferative capacity. It suggested that the harvested cells in this study might be feline UC-MSCs, which could proceed to the next step of identification.

The surface markers for defining MSCs in animals have not been established as they are in human (21). According to the results reported in the literature about feline MSCs isolated from different tissues: adipose, amniotic fluid, and bone marrow, feline MSCs basically all express CD44 and CD90, but lack the expression of CD34, while other expression markers are different in various studies (12, 21–23). Our study showed that most of the feline UC-MSCs expressed MSCs surface markers CD44 and CD90, and lacked the expression of hematopoietic markers (CD34 and CD45).

Differentiation capacity is another important feature of MSCs. At present, it is widely known that BM-MSCs and UC-MSCs are involved in many physiological processes, whose differentiation potential plays a critical role in tissue repair, wound healing, and regenerative medicine (24, 25). Many researchers support the hypothesis that MSCs and fibroblasts are indistinguishable in terms of morphology, cell surface markers, immunologic properties, and differentiation potential, whereas according to some evidences MSCs retain a multipotent differentiation capacity, but fibroblasts seem to display only limited, or no such multipotent differentiation (26). The distinction among these cell types in terms of ability to differentiate is also crucial to recognize specific cell precursor at the origin of many pet diseases. For the first time, we compared the differentiation potential of three types of cells, among which C3H10T1/2 as a positive control and feline fibroblasts as a negative control, with the same differentiation media and methods used for the osteogenic and adipogenic induction. Both C3H10T1/2 and feline UC-MSCs exhibited similar mineral deposits and lipid droplets, while fibroblasts isolated from feline did not differentiate toward the osteogenic or adipogenic lineage. One possible reason for the difference in differentiation ability between feline UC-MSCs and feline fibroblasts might be that MSCs populations are more intrinsically heterogeneous than fibroblasts (27), which contributes to their multi-directional differentiation potential. Although feline UC-MSCs were able to differentiable into not only osteocytes but also adipocytes, there were less stained areas in osteogenic and adipogenic differentiation than in C3H10T1/2. It suggested that the optimal differentiation induction system of MSCs derived from different species and different parts was discrepant, which was confirmed by our subsequent optimized system of osteogenic and adipogenic induction for feline UC-MSCs.

In previous studies, differentiation potential was shown to depend on various factors, including tissue of origin and concentration of inducible factors (28–30). In the study, we explored the optimal differentiation conditions of feline UC-MSCs and further understand the differentiation mechanism. Previous study demonstrated that alkaline phosphatase (ALP) activity and ability to form mineralized deposits were higher in MEM cultures than in DMEM cultures (31). Zainal et al. and Salehinejad et al. also revealed that α-MEM supported the expansion and differentiation of MSCs more strongly than DMEM (32, 33). So, we decided to use MEM instead of DMEM in the optimized osteogenic and adipogenic medium. Moreover, among osteogenic agents, the differentiation factors such as L-ascorbate acid phosphate and β-glycerophosphate are widely used in cell differentiation (34). β-glycerophosphate is closely associated with osteogenic signaling and mineralization of cells (35) and L-ascorbate acid phosphate is vital for MSCs differentiation and bone formation (36). Therefore, we also enhanced L-ascorbate acid phosphate and β-glycerophosphate concentrations in the optimized osteogenic medium. Results from phase contrast microscopy revealed that more nodule formation was observed preferentially at day 14 post-differentiation induction when compared to the previous osteogenic medium. Dexamethasone is a glucocorticoid hormone and an important regulator of lipid differentiation of mesenchymal stem cells (37). Our results showed that the optimized adipogenic medium with dexamethasone significantly stimulated the accumulation of lipid droplets. These results are in line with those previously published studies, showing that dexamethasone contributes to the increased adipocyte number and lipid accumulation (38). In conclusion, the optimal condition reduced the induction time and showed more efficient osteogenic and adipogenic differentiation ability as compared to the previous condition in feline UC-MSCs, which should be helpful in the development of stem cell therapy.

### 4.2. Antioxidant capacity and mechanism of feline UC-MSCs

It is known that the antioxidant and immunomodulatory functions of MSCs through paracrine are very important for their application in regenerative medicine. *In vitro*, MSCs can reduce oxidative stress-induced injury in cardiomyocytes, renal cells, endothelial cells, immune cells, hepatocytes, islet cells, fibroblasts, and skeletal muscle (3). According to these studies, MSCs have antioxidant properties by scavenging free radicals directly, promoting endogenous antioxidant defenses, and suppressing the immune system to prevent oxidative injury (3). Although MSC treatments are unequivocally proven to reduce levels of oxidative stress in cells and animal models, most of the data come from human and mouse studies. Currently, the antioxidant role and mechanism in feline UC-MSCs has not been reported. H_2_O_2_ is often used as a model drug for oxidative damage due to its relatively stable properties *in vitro* (39). Therefore, feline UC-MSCs and feline fibroblasts were modeled with a certain concentration of H_2_O_2_ to compare their antioxidant capacity in this study. ROS as a signal molecule supports normal physiological activities, but the excessive ROS will cause cell death and oxidative stress (40). MDA is one of the most frequently measured biomarkers of oxidative stress, and generally, the increase of MDA content indicates the degree of oxidative stress damage of cytoplasmic membrane (41). In the present study, the treatment with H_2_O_2_ significantly increased the levels of ROS and MDA in feline UC-MSCs and feline fibroblasts throughout of 8 h, which indicated that oxidative stress was induced. It was worthy to note that the results of morphological change and cell viability indicated that feline UC-MSCs had higher resistance to H_2_O_2_ compared with feline fibroblasts, which might be related to the stronger enhancement of antioxidant capacity in MSCs by the pretreatment with a certain concentration of H_2_O_2_ (42). SOD and GSH-Px are the main antioxidant enzymes that scavenge ROS, protecting against ROS-induced damage (43). Several recent studies suggested that MSCs can upregulate the levels of antioxidant enzymes SOD1, SOD2, and GSH-Px *in vitro*, thus ensuring their tolerance to oxidative environments or inflammatory injury (3, 44–46). The activities of SOD2 or/and GSH-Px showed significantly increase in feline UC-MSCs when treated with 100 μM and 300 μM H_2_O_2_ and the CAT activity of feline UC-MSCs was higher than that of feline fibroblasts in the control group and 100 μM H_2_O_2_-treated group, which might contribute to the greater antioxidant capacity of feline UC-MSCs than feline fibroblasts. One of the main functions for SOD is to scavenge O_2_^−^ and transform it into H_2_O_2_ (47), which might explain the decrease of SOD2 activity in feline UC-MSCs after treated with 100 μM H_2_O_2_ via feedback mechanism. Although the main functions of both CAT and GSH-Px are to neutralize H_2_O_2_ (48), the activity of CAT did not increase in feline UC-MSCs after treated with 100 μM and 300 μM H_2_O_2_, which suggested that the GSH-Px might be important for the antioxidant response of feline UC-MSCs. Surprisingly, there was no change for the level of MDA and the activities of SOD2 and GSH-Px while the activity of CAT decreases in 500 μM H_2_O_2_-treated feline UC-MSCs, which suggested that the other antioxidant mechanisms and the consequent feedback roles might be existed.

Given the desirable antioxidant properties of MSCs, whose mechanisms are usually associated with numerous oxidant pathways, such as Nrf2/HO-1, MAPK, and NF-κB signaling pathway (49–51), we tested the effects of NF-κB inhibitor BAY 11-7,082 on the H_2_O_2_-induced morphological changes of feline UC-MSCs. The results suggested that NF-κB pathway was involved in the antioxidative process of feline UC-MSCs. The subsequent results of qRT-PCR and Western Blot showed that the NF-κB pathway was activated in feline UC-MSCs in response to H_2_O_2_ stimuli. NF-κB can be activated when its inhibitor, IκB, is phosphorylated and degraded from the cytoplasm (52). We found an obvious effect of H_2_O_2_ on p-IκB protein levels in feline UC-MSCs, and the significant increases of p-IκB were reversed after the addition of BAY 11-7,082. Activated NF-κB forms a variety of homo- or heterodimers with transcriptional activity, of which p65 and p50 are the most common (53). We did not monitor the mRNA expression level of p65, which was consistent with the results of Western Blot. However, the increases of p-p50 protein were observed with H_2_O_2_ stimulation, indicating the subsequent potential translocation into the nucleus, whereas BAY 11-7,082 reversed the change, indicating that the phosphorylation activation of p50 was caused by IκB phosphorylation/degradation. The changes of IκB and p50 mRNA/protein induced by 500 μM H_2_O_2_ were basically upregulated except for IκB mRNA and were reversed by BAY 11-7,082, but irregular in 100 μM or 300 μM H_2_O_2_ group, suggesting that the involved mechanism was complicated, which needed to be further investigated. NF-κB signaling pathway can activate antioxidant genes, express antioxidant proteins and maintain the redox environment of cells (54). This expressional upregulation of MnSOD and FHC mRNA/protein in H_2_O_2_-treated group indicated that the activated p-p50 might be an early response to oxidative stress to protect cells via upregulating the downstream antioxidant targets in feline UC-MSCs. Under stress conditions, MnSOD and FHC play an integral role in antioxidant activity. MnSOD converts superoxide radicals into oxygen and hydrogen peroxide, whereas ferritin minimizes iron-catalyzed ROS production from hydrogen peroxide by binding free iron (19). The effects of H_2_O_2_ on the MnSOD and FHC mRNA/protein were weakened to different degrees by inhibitor-BAY 11-7,082, which confirmed the regulation relationship of p-p50 and both of them in feline UC-MSCs. Combining these results, NF-κB signaling pathway plays a crucial role in the response of feline UC-MSCs to H_2_O_2_ stimulus, which shed new light on the antioxidation mechanism in feline UC-MSCs. Certainly, in practical application, we must consider that the downstream target genes of NF-κB also include inflammatory factors, although studies have shown that MSCs can secrete anti-inflammatory cytokines. Therefore, a holistic study is needed to find the right balance and further formulate the use stage and dosage of MSCs in clinical application.

## 5. Conclusion

**Figure 13:**
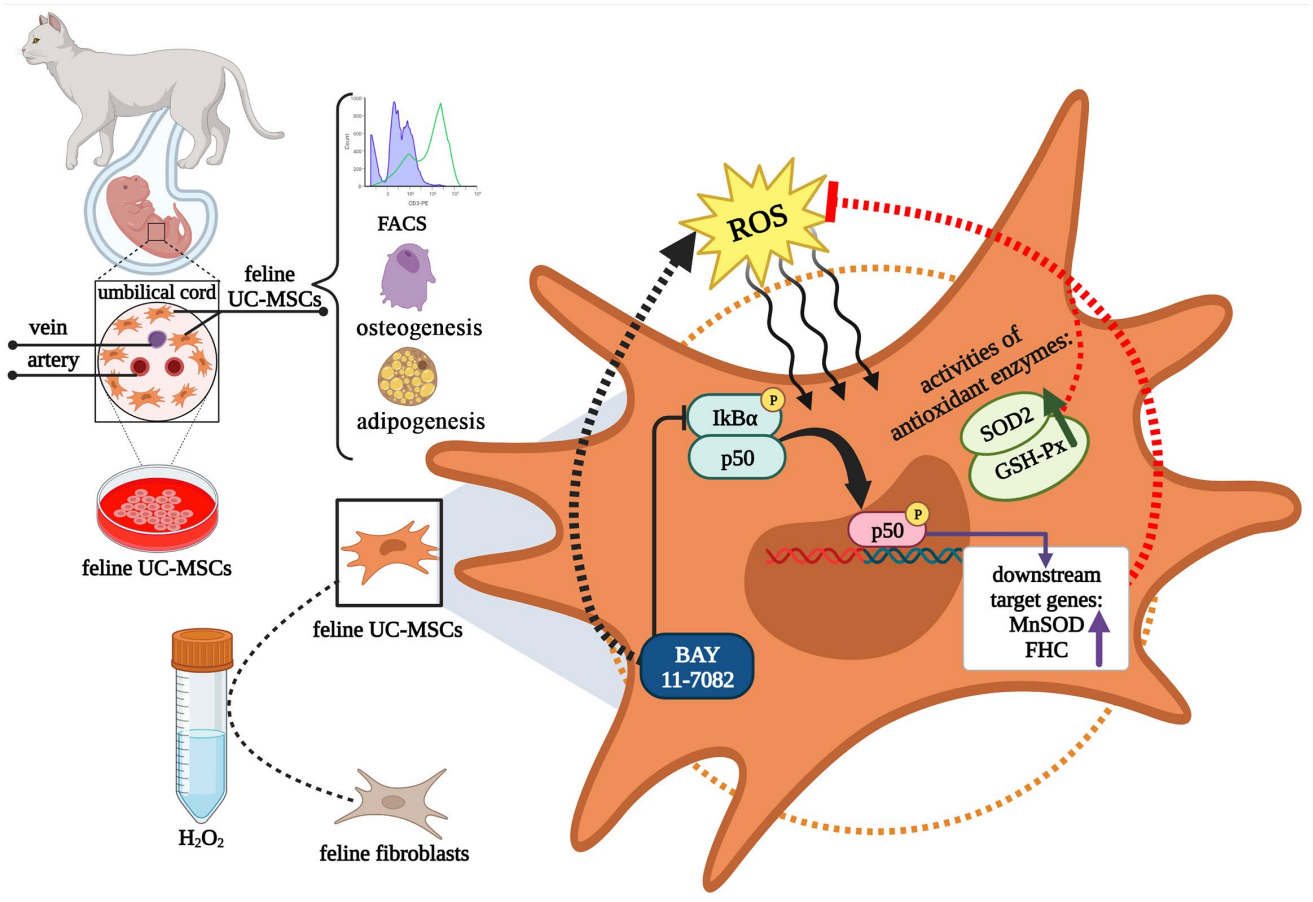
Schematic diagram illustrating the isolation and identification of feline umbilical cord-derived mesenchymal stem cells (UC-MSCs), and antioxidant mechanism by NF-κB signaling pathway under H_2_O_2_ stimulation. This graphic was created with BioRender.com.

In summary, we successfully isolated and identified mesenchymal stem cells derived from feline umbilical cord, and optimized the osteogenic and adipogenic induction system for feline UC-MSCs. Feline UC-MSCs exhibited greater anti-oxidative capacity compared with feline fibroblasts upon H_2_O_2_ treatment. The antioxidant effect of feline UC-MSCs depended at least in part on the activation of NF-κB signaling pathway (Figure 13). These findings may provide important evidence for elucidating the antioxidant mechanism of UC-MSCs and promoting the clinical application of feline UC-MSCs.

## Data availability statement

The raw data supporting the conclusions of this article will be made available by the authors, without undue reservation.

## Ethics statement

The animal study was reviewed and approved by the Committee for the Care and Use of Experimental Animals, Shanxi Agricultural University, Shanxi, China. Written informed consent was obtained from the owners for the participation of their animals in this study.

## Author contributions

Z-HZ, RD, and JQ designed all the experiments. Z-HZ, ZY, JA, and D-PZ performed the isolation and treatment of feline UC-MSCs and feline fibroblasts. Z-HZ, JL, H-JW, M-MD, and JY performed the experiment of qRT-PCR and WB. Z-HZ and YC performed the experiment of flow cytometry. Z-HZ analyzed the data and wrote the manuscript. RD and JQ revised the manuscript. All authors contributed to the article and approved the submitted version.

## Funding

This work was supported by Fundamental Research Program of Shanxi Province (20210302123394); Innovation Project for Graduate Students in Shanxi Province (2021Y348); Research Project Supported by Shanxi Scholarship Council of China (2022-102); National Natural Science Foundation of China (32272964 and 31872438); Fund Program for the Scientific Activities of Selected Returned Overseas Professionals in Shanxi Province; Program for the Top Young and Middle-aged Innovative Talents of Shanxi Agricultural University (BJRC201204); Horizontal Science and Technology Project of Shanxi Agricultural University (2015HX12).

## Conflict of interest

The authors declare that the research was conducted in the absence of any commercial or financial relationships that could be construed as a potential conflict of interest.

## Publisher’s note

All claims expressed in this article are solely those of the authors and do not necessarily represent those of their affiliated organizations, or those of the publisher, the editors and the reviewers. Any product that may be evaluated in this article, or claim that may be made by its manufacturer, is not guaranteed or endorsed by the publisher.
